# HPV 16 detection in cervical lesions, physical state of viral DNA and changes in p53 gene

**DOI:** 10.1590/S1516-31802003000200007

**Published:** 2003-03-05

**Authors:** Ledy do Horto dos Santos Oliveira, Eliane de Vasconcelos Machado Rodrigues, Ana Paula Terra Alvim de Salles Lopes, André de Paula Fernandez, Silvia Maria Baeta Cavalcanti

**Keywords:** HPV, Viral, DNA, Integration, Gene, Cervical, Lesions, HPV, Integração, DNA Viral, Gene, Lesões Cervicais

## Abstract

**CONTEXT:**

Persistent infection with high risk human papillomavirus (HPV) has been linked to cervical carcinoma. Integration of viral DNA into host cell DNA is essential for this cancer development, promoting disruption of the HPV E2 gene, thus leading to unregulated increases in E6 and E7 proteins and inactivating the products of p53 and Rb tumor suppressor genes.

**OBJECTIVE:**

To investigate HPV 16 infection in cervical lesions, physical state of viral DNA and p53 gene alterations in a group of women attending a public health service.

**DESIGN:**

Prospective, non-controlled, transversal study.

**SETTING:**

Gynecological clinic of the School od Medicine, Universidade Federal Fluminense.

**SAMPLE:**

43 consective patients with cervical lesions referred to our service.

**MAIN MEASUREMENTS:**

Cases were classified via cytology/histology as normal, HPV infection, condyloma, low-grade squamous intraepithelial lesion (LSIL), high-grade squamous intraepithelial lesion (HSIL) and carcinoma. HPV infection was studied via polymerase chain reaction (PCR) using two PCR primer sets, to determine DNA integration. p53 gene changes were investigated by single-strand conformation polymorphism (SSCP) analysis.

**RESULTS:**

One normal case, 7 HPV infections, 6 condylomas, 7 LSIL, 14 HSIL and 8 cancers were found, with 95% positive for HPV genome when tested using both L1 and E6 primers. HPV 16 was most prevalent (73.1%). HPV 16 DNA was integrated within the host genome in 3 LSIL. One LSIL progressed to HSIL by 13 months after first diagnosis. Among HPV 16-positive HSIL cases, 50% contained integrated viral DNA. HPV 16 E2 gene disruption was seen in 7 cancers (87.5%). Only smal-cell carcinoma showed intact HPV 16 E2 gene. Abnormal p53 bands detected by PCR/SSCP were observed in 4 cases: 2 squamous carcinoma with parametrium (exon 8) and two cervical intraepithelial neoplasia (CIN) III (exons 5 and 7). All cases presented HPV 16 E2 gene loss.

**CONCLUSIONS:**

The sample had a high rate of high-risk HPV detected in benign and malignant lesions; high cervical cancer burden; HPV 16 DNA integration in all except one case of cancer; p53 gene changes in CIN III and in invasive cancer cases associated with DNA integration.

## RESUMO

**CONTEXTO:**

A infecção persistente por papilomavírus humano (HPV) de alto risco tem sido associada ao carcinoma cervical. Uma fase obrigatória no desenvolvimento deste câncer é a integração do DNA viral ao DNA das células hospedeiras. A integração viral promove a degradação do gene E2 do papilomavírus humano e leva a um aumento descontrolado das proteínas E6 e E7. Estas proteínas virais de HPVs oncogênicos inativam os produtos dos genes supressores de tumor p53 e Rb. Inativação da proteína p53 é o resultado de vários fatores incluindo mutação no gene para p53.

**OBJETIVO:**

Investigar lesões cervicais associadas à infecção por papilomavírus humano, o estado físico do DNA viral e alterações no gene para p53 em um grupo de mulheres atendidas em um serviço público de saúde.

**TIPO DE ESTUDO:**

prospectivo, não-controlado.

**LOCAL:**

Serviço de Patologia Cervical, Ambulatório de Ginecologia da Faculdade de Medicina, Universidade Federal Fluminense.

**PARTICIPANTES:**

43 mulheres atendidas em consulta ambulatorial de rotina para prevenção às lesões cervicais.

**VARIÁVEIS ESTUDADAS:**

Pela citologia/histologia os casos foram classificados como normal, infecção por papilomavírus humano, condiloma, lesão cervical de baixo graus, lesão cervical de alto grau e carcinoma. A presença de papilomavírus humano foi detectada por reação de polimerase em cadeia (PCR) utilizando-se dois pares de oligonucleotídeos. Para determinar a integração do DNA viral, usaram-se oligonucleotídeos específicos para o gene E2 do tipo HPV 16 que amplificam um fragmento de 1.026 pares de base. Alterações no gene para p53 foram investigadas por polimorfismo conformacional da fita simples.

**RESULTADOS:**

Foi detectado um caso de cérvice normal, sete infecções por papilomavírus humano, seis condilomas, sete lesões cervicais de baixo graus, 14 de alto grau e oito carcinomas. O estudo revelou que 14 mulheres sem queixas de doença genital eram portadoras de lesões de vários tipos incluindo três lesões cervicais de alto grau e um caso de carcinoma *in situ*. 95% das pacientes foram positivas para a presença do genoma de HPV quando testadas para os genes L1 e E6, com prevalência do HPV tipo 16 (73.1%). Detectamos DNA do HPV 16 integrado ao genoma hospedeiro em três dos sete casos de lesões cervicais de baixo grau. Entretanto, um destes pacientes progrediu a lesão cervical de alto grau 13 meses após o primeiro diagnóstico. Entre os casos de lesão cervical de alto grau positivos para HPV 16, 50% continham o DNA viral no estado integrado foi encontrado. A falta do gene E2 do HPV 16 foi observada em sete dos carcinomas (87.5%) e somente no carcinoma de células pequenas foi detectado o gene E2. Bandas anormais do gene p53 detectadas por PCR/SSCP foram observadas em quatro casos: dois carcinomas escamosos com envolvimento do paramétrio (exon 8) e dois casos de NIC III (exons 5 e 7, respectivamente). Todos os casos apresentaram perda do gene E2 do HPV 16.

**CONCLUSÕES:**

A amostra analisada tinha alta taxa de HPV de alto risco detectada tanto em lesões benignas como em lesões malignas; alto índice de câncer cervical; integração do DNA do HPV 16 em quase todos os casos de câncer com uma exceção; alterações no gene para p53 em NIC III e em câncer invasivo associadas à integração do DNA.

## INTRODUCTION

In the last decade, breast cancer has become the highest cause of morbidity and mortality among female malignancies, but cervical cancer represents the leading cause of cancer-related deaths among women in developing countries, including Brazil.^1^ Nearly 40,000 cases of cervical cancer arise among Brazilian women every year.^2^ Lopes et al., studying the behavior of Brazilian women in the light of cervical cancer prevention, verified that most people had not undergone preventive examinations.^3^ Uterine cervical cancer and non-specific uterine cancer combined are the second greatest underlying cause of death due to neoplasms for women aged 30-49 in the state of São Paulo.^4^

Evidence from laboratory and epidemio-logical studies has shown an association between human papillomavirus (HPV) infection and both cervical cancer and pre-cancerous neoplasias.^5,6^ High-risk HPV types like HPV 16 and 18 have been strongly linked to cervical carcinoma.^7^ In addition to HPV 16 being common in the general population, it remains among the most prevalent individual type in cervical neoplasias.^8^

High-risk HPVs can integrate into host cell DNA, and this is essential data regarding cancer development. Viral integration promotes the disruption of the HPV E2 gene leading to unregulated increases in the E6 and E7 proteins.^9^ These viral proteins of oncogenic HPVs inactivate the products of p53 and Rb tumor suppressor genes, respectively. The tumor suppressor gene functions include regulation of the cell cycle and the cellular response to DNA damage, initiation of DNA repair and replication, induction of apoptosis and promotion of cell differentiation.^10^ Inactivation of the p53 tumor suppressor gene is correlated to a critical step in the development of many human cancers.^11^ It may result from a number of events including mutation of the p53 gene (with or without associated allelic deletions) and binding of the p53 to cellular or viral proteins. In cervical carcinoma, loss of p53 function can occur by interaction with E6 protein of high-risk HPV types.^12^

In this work, we conducted a study on a group of women referred to our public health service for assessment of cytological/histological abnormalities in genital lesions. We detected HPV type 16 infection, the physical status of viral DNA and p53 gene alterations.

## METHODS

We studied 43 consecutive women attended at Hospital Universitário Antônio Pedro of the Universidade Federal Fluminense, Rio de Janeiro, between April 2000 and June 2002. These women have litlle or no access to routine, annual Papanicolaou exams in local services and were referred to our hospital for investigation of different kinds of cervical lesions. Colpocytology test screening was performed at the first or subsequent visit to our clinic. The age range was 18-68 years with an average of 37.8 years, with a standard deviation of 11.9.

Two cervical smears containing ectocervical and endocervical cells were taken from each patient, one of them placed in TE buffer (Tris 10 mM pH 7.4, EDTA 1mM — ethylene diamine tetraacetic acid — 1 mM) and stored at −20° C and the other destined to Papanicolaou exam, by three examiners per slide. For women with abnormal cervical cytology, biopsies were performed.

The cases were then classified by our Service as normal, HPV infection, low-grade squamous intraepithelial lesions (LSIL - CIN I, cervical intraepithelial neoplasia), high-grade squamous intraepithelial lesions (HSIL - CIN II and III), and carcinoma (*in situ* carcinoma, squamous invasive carcinoma, adenocarcinoma, small-cell carcinoma).

Samples were incubated for 4 hours at 50° C in 200 ml of digestion buffer (10 mM Trishydrochloric acid pH 8.3, 1 mM EDTA pH 8.0, 0.5% Tween 20, proteinase K; final concentration of 400 μg/ml). Later, they were extracted with phenol-chloroform-isoamyl alcohol (25:24:1). DNA was precipitated with one-tenth volume of 0.3 M sodium acetate and three volumes of 100% ice-cold ethanol, washed with 70% ethanol, air-dried and suspended in 50 μl of sterile water.

MY09/11 consensus primers, which amplify 450-bp (base pair) DNA sequences within the L1 region of HPV, were used to detect generic HPV DNA. Amplification was carried out in 50 μl of reaction mixture (1 X polymerase chain reaction [PCR] buffer, 200 mM dNTPs, 1.5 mM MgCl_2_, 50 pmol of each primer, 0.25 U unit of Taq polymerase, and 5 μl of sample) with 35 cycles of amplification. Each cycle included a denaturation step at 94° C for 1 minute, an annealing step at 55° C for 2 minutes, and a chain elongation step at 72° C for 2 minutes using DNA Thermal Cycler (Perkin Elmer, CETUS). The beta-actin primers (0.1 pmol each), which amplify a 330-bp region of the human DNA, were used as an internal control. Polymerase chain reaction products were analyzed on 1.3% agarose gel with ethidium bromide staining for visualization of DNA under ultraviolet light and their molecular weight was determined by comparison with a 100-bp DNA ladder.

Human papillomavirus typing was done by polymerase chain reaction amplification using primers from the E6 gene DNA sequences of HPVs 6, 11, 16, 18, 31, 33, and 35. These primers yielded 230, 89, 134, 119, 97, 132 and 186-bp fragments, respectively.^13^ The PCR included steps at 94° C for 30 seconds, 60° C for 1 minute, and 72° C for 1 minute. Negative controls for background contamination did not add to the DNA template. The polymerase chain reaction run was completed by extension for 10 minutes at 72° C.

HPV 16 E2 type-specific primers, which amplify 1026-bp fragments, were used to determine DNA integration. The following primers were used: sense 2810-5’ ATGAAAATGATAGTACAGAC-2819 and antisense 3836-5’ CCAGTAGACACTGTAAATAG-3818.^14^ Absence of the E2 gene was considered to be a sign of E2 region disruption. The PCR was done as above.

The primers used for polymerase chain reaction amplification of p53 gene exons 5 to 8 and resulting product sizes are given in Table 1. The PCR was carried out in a volume of 20 μl consisting of 1 X buffer, (Tris-HCl pH 8.0, 50 mM KCl), 50 μM dNTPs, 1.5 mM MgCl_2_, 1 μM of each primer, 0.25 U unit of Taq polymerase, and 1 ml of sample. It was hot-started by the addition of the reaction mixture at 94° C, 35 cycles of 30 seconds at 94° C, 1 minute at 60° C, and 1 minute at 72° C. The polymerase chain reaction amplification was completed by extension for 10 minutes at 72° C. The positive controls were DNA from tumor specimens, whereas negative control for background contamination did not add to the DNA template. Extracted DNA from a benign wart was used as a normal control. Polymerase chain reaction products were visualized by electrophoresis performed at room temperature for 3 hours at 30 mA. Single-strand DNA for single-strand conformation polymorphism (SSCP) analysis was produced by combining equal volumes of PCR product and formamide-loading buffer (95% formamide, 10 mM EDTA, 0.05% bromophenol blue, 0.05 xylene cyanol) and heating at 95°C, for 10 minutes. The reaction was left on ice until submitting to electrophoresis. Non-denaturing polyacrylamide gel (49:1 acrylamide-bisacrylamide ratio) was used. After the run, the gel was fixed in 7.5% acetic acid, washed, and silver-stained. Briefly, the gel was soaked in 10% ethanol and 1% nitric acid, and immersed in impregnation solution, visualized in developing solution, and fixed in 10% acetic acid.

**Table 1:** Primer sequences and polymerase chain reaction (PCR) product sizes for p53 exons 5 to 8

Exon	Nucleotide sequence (5’- 3’)	PCR product size (bp)
5	TGT TCA CTT GTG CCC TGA CT	310
	AGC AAT CAG TGA GGA ATC AG	
6	TGG TTG CCC AGG GTC CCC AG	223
	TGG AGG GCC ACT GAC AAC CA	
7	CTT GCC ACA GGT CTC CCC AA	248
	AGG GGT CAG CGG CAA GCA GA	
8	TTG GGA GTA GAT GGA GCC T	313
	AGA GGC AAG GAA AGG TGA TA	

## RESULTS

In accordance with the cytological/histo-logical diagnosis, we found one normal case, seven HPV infections, six condylomas, seven lLSIL, fourteen HSIL and eight carcinomas. The average age of patients with malignant lesions was about 45 years. Among the 43 women, six (14%) were also infected with the human immunodeficiency virus (HIV). The results are summarized in Table 2.

**Table 2:** Cytological/histological findings associated with human papillomavirus (HPV) types

Cytology/histology	HPV types									
	6	11	16	18	33	6,11	6,16	6,33	16,18	16,33
Normal (N=1)				1						
				***						
HPV (N=7)*			5				1			
			***							
Condyloma (N=6)			2			1	1	1	1	
LSIL (N=7)	2		3		1			1		
			***		***					
HSIL (N=14)**			9	1			1		1	
			****				***			
Carcinoma (N=8)			5						2	1
**Total = 43**	**2**		**24**	**2**	**1**	**1**	**3**	**2**	**4**	**1**

* *One type not identified;*

** *HPV infection not detected in two cases;*

*** *One HIV-positive case;*

**** *One HIV-positive case progressed to cancer.*

Ninety-five percent of the patients (41) were positive for the presence of HPV genome when they were tested using both L1 and E6 primers. Out of 43 samples, 67.4% (29) were positive for at least one virus type and 25.5% (11) presented infections by multiple types. Out of six cases of condyloma, four were associated with squamous intraepithelial lesion and all of them were positive to HPV. The study revealed that fourteen women had no complaint but had several kinds of cervical lesions including three HSIL and one *in situ* carcinoma. All except one were HPV-infected. HPV 16 was the most prevalent type (60.4%) in single infections and both single and multiple infections (73.1%). Thus, low-risk types were detected in 18.6%, HPV 18 in 14% and HPV type 33 in 9% of the cases. HPV 16 was found in 100% of the carcinomas alone or in association with type 18 or 33.

The HPV 16 DNA appeared also to be integrated within the host genome in three out of seven low-grade squamous intraepithelial lesions. However, one of these patients had progressed to HSIL by thirteen months after the first diagnosis. Among high-grade squamous intraepithelial lesion cases positive to HPV 16, 50% (7/14) contained viral DNA in the integrated state. Disruption of the 16 E2 gene was seen in all seven cancer cases (87.5%) whereas only the small-cell carcinoma showed intact 16 E2 gene (Table 3).

**Table 3:** Human papillomavirus (HPV) detection in carcinoma cases and Type 16 E2 integration

No of case	Age	Histological type	HPV types	16 E2 integrity
3	41	Squamous carcinoma	16,18	No
15	38	*In situ* adenocarcinoma	16,3	No
21	37	Adenocarcinoma/HPV	16	No
23	33	Squamous carcinoma	16	No
28	43	Squamous carcinoma/HPV	16	No
30	68	Small cell carcinoma	16	Yes
33	57	*In situ* carcinoma	16	No
38	40	*In situ* carcinoma	16,18	No

**Figure 1:**
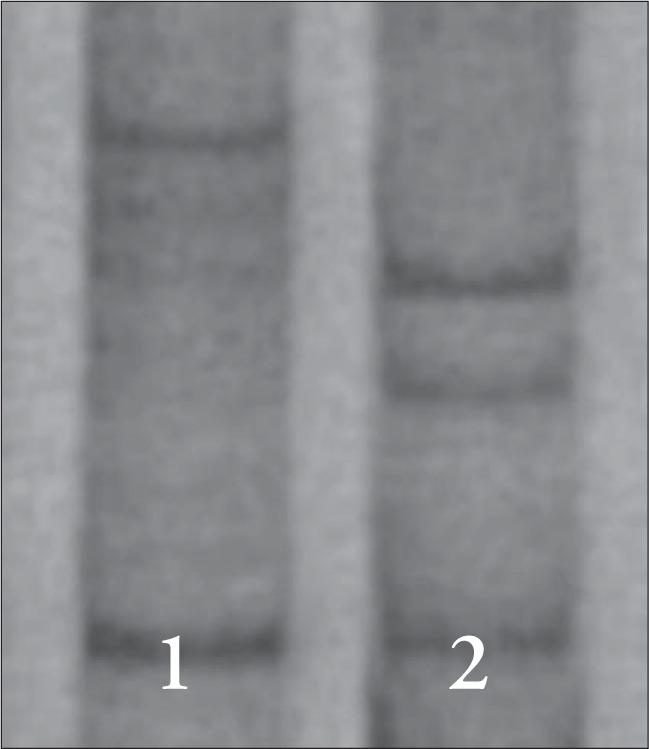
Screening of p53 mutation on exon 5 by polymer-ase chain reaction/single-strand conformation polymorphism. Number 1: normal run. Number 2: abnormal band. (CIN III).

Abnormal bands of p53 gene were observed in four cases: two squamous carcinoma with parametrium involvement (exon 8) and two CIN III (exons 5 and 7, respectively, Figure 1). One carcinoma had poor prognosis after 24 months. All cases presented loss of the HPV 16 E2 gene.

The persistence of HPV 16 was seen in four out of five patients treated for CIN III or *in situ* carcinoma following conization. To verify persistent HPV infection, the PCR test was done when the patients came back for routine follow-up three months after surgery. Only one *in situ* carcinoma case had the viral clearance demonstrated. One case (CIN III) infected with HPV 16 and 18 had their HPV 16 but not HPV 18 cleared. All patients had their borderline tissue freed of abnormal cells. After two years, three out of four HPV 16 infected patients did not present recurrence of the lesions. However, one HIV seropositive woman who presented CIN III progressed to invasive carcinoma 11 months after surgery.

## DISCUSSION

The main findings of this prospective study were: HPV DNA identified in 95% of all cases; a high rate (86%) of high-risk HPV detected in benign as well as in malignant lesions; high burden of cervical cancer; HPV 16 DNA integration in all except one case of cancer; p53 gene changes in CIN III; and invasive cancer cases associated with DNA integration.

Infection with high-risk human papillomavirus types is frequent among sexually active women, with incidence ranging from 15 to 40%.^2^ However, the majority of the infections are found to be transient because most individuals develop a specific immune response.^15^ When the infection persists, pre-cancer lesions may develop. About 1% of the general population present genital warts and 4% of all women have cervical precancerous lesions.^16^

In the population studied, human papillomavirus presence is associated with 85,7% high-grade squamous intraepithelial lesion and 100% carcinoma. The mean age of patients with malignant lesions was about 45 years, considered low in comparison with developed countries. In spite of the random feature in the enrollment of the patients, the women in this sample came from lower-income classes. Moreover, the Hospital Universitário Antônio Pedro is the main general hospital in a large densely populated geographical area. The lack of public healthcare clinics for providing appropriate attendance of severe dysplasia among women and also for its adequate treatment oblige this hospital to receive a high number of these cases. Therefore, these are women with little or no access to annual Papanicolaou exams that would detect inflammatory or pre-neoplastic lesions (rather than neoplastic lesions) and prevent their malignant evolution. This, in association with other cofactors, may contribute to the disproportionately high rate of pre-cancerous and cancerous lesions observed in our sample. Lack of access to healthcare, poor nutrition, multiple parities, human immunodeficiency virus (HIV) and other sexually transmitted diseases (STD) also increase the susceptibility to cervical carcino-genesis in our population.

Another remarkable finding was the high rate of HIV-positive women who were infected with human papillomavirus. It is an indication of the misinformation about health monitoring among poor Brazilian people. Heterosexual transmission without using a condom is the main route to female human immuno-deficiency virus infection. The presence of other sexually transmitted diseases, such as human papillomavirus infection, promotes breaks in the genital tract and increases the susceptibility to acquiring the human immunodeficiency virus.^17^

In an extensive molecular epidemiological study, Villa et al.^18^ found that infections with non-European variants of HPV 16 and 18 had a general tendency to persist more frequently and to be more associated with pre-invasive lesions. In their analysis, 33% (15/46) women infected with European variants were non-white as compared to 58% (11/19) infected with non-European variants in the same ethnic category. It is worth noting that out of the eight cancer cases in our sample, seven (87.5%) were non-white women. It may be possible that, in addition to the co-factors above mentioned, non-European variants of HPV 16 harbored in non-white patients have contributed to increasing the rate of cervical cancer in this study.

In most cervical immortalized cells, high-risk HPV DNA often integrates into the cellular genome. The integration is processed throughout the E2 gene by disrupting some part of it and causing overexpression of the E6 and E7 proteins. This results in the loss of anti-oncogenic function in p53 and Rb proteins. On the basis of these events, we investigated the physical state of HPV 16 DNA using the polymerase chain reaction assay. The result was no different from other reports.^9,19,20^ We found loss of the E2 gene in 42.8% of low-grade squamous intraepithelial lesion and in 50% of high-grade squamous intraepithelial lesions. One low-grade squamous intraepithelial lesion case had progressed to high-grade squamous intraepithelial lesion by thirteen months after the first diagnosis. These data support the hypothesis that integration mainly happens at an early stage in the development of cervical neoplasia. It is possible that the increased expression of the E6 and E7 proteins may result in a selective growth advantage over cells harboring individualized HPV 16 DNA.

In a study on Korean women, 92.2% of HPV 16 cancers revealed pure or mixed forms of integrated DNA.^14^ We found a lack of E2 gene in 87.5% (7/8) of the carcinomas. The intact E2 gene form was only seen in small-cell carcinoma. This has also been observed by other researchers.^14^

We detected p53 gene changes in four cases (14% of the CIN III and 25% of the *in situ* carcinoma). All of them contained integrated HPV 16 DNA, thus showing that human papillomavirus infection and p53 gene alterations are not excluding events for cancer development. In a study to determine p53 mutation by polymerase chain reaction/single-strand conformation polymorphism and sequencing techniques, point mutation was detected in 11% (5/46) of HPV-infected cervical carcinoma and no mutation was found in two HPV-negative cases.^21^ The rapid progress of squamous invasive cancer observed in our sample may suggest a synergic effect of the two events. However, the low frequency of the p53 gene changes (19% CIN III/*in situ* carcinoma) in comparison with the high frequency of high-risk human papillomavirus infection (85.7% CIN III/*in situ* carcinoma) strengthens the view that p53 inactivation by human papillomavirus proteins plays a major role in the pathogenesis of cervical cancer.

Human papillomavirus detection following conization even when CIN III and *in situ* carcinoma were successfully treated is more likely to result in viral persistence than a new infection. However, the residual human papillomavirus did not promote recurrent disease over a two-year period in most of the women surgically treated. The obvious immune failure of one HIV-seropositive patient allowed the recurrence of the disease and its progression to invasive cancer.

Although cervical cancer is a preventable disease, the lack of an effective public healthcare police hinders the reduction of this malignancy among low-income women in particular.
